# Use of smartphone-based remote assessments of multiple sclerosis in Floodlight Open, a global, prospective, open-access study

**DOI:** 10.1038/s41598-023-49299-4

**Published:** 2024-01-02

**Authors:** Jiwon Oh, Luca Capezzuto, Lito Kriara, Jens Schjodt-Eriksen, Johan van Beek, Corrado Bernasconi, Xavier Montalban, Helmut Butzkueven, Ludwig Kappos, Gavin Giovannoni, Riley Bove, Laura Julian, Mike Baker, Christian Gossens, Michael Lindemann

**Affiliations:** 1 Division of Neurology, St. Michael’s Hospital, University of Toronto, Toronto, ON Canada; 2 F. Hoffmann-La Roche Ltd., Basel, Switzerland; 3 Department of Neurology–Neuroimmunology, Centre d’Esclerosi Múltiple de Catalunya (Cemcat), Hospital Universitari Vall d’Hebron, Barcelona, Spain; 4 Department of Neuroscience, Central Clinical School, Monash University, Melbourne, Australia; 5 Research Center Clinical Neuroimmunology and Neuroscience Basel (RC2NB), University Hospital Basel, University of Basel, Basel, Switzerland; 6 Queen Mary University of London, London, UK; 7 Department of Neurology, UCSF Weill Institute for Neurosciences, University of California San Francisco, San Francisco, CA USA; 8 Genentech, Inc., South San Francisco, CA USA; 9 Present Address: Biogen Digital Health International GmbH, Baar, Switzerland; 10 Present Address: Limites Medical Research Ltd., Vacallo, Switzerland

**Keywords:** Multiple sclerosis, Outcomes research

## Abstract

Floodlight Open was a global, open-access, digital-only study designed to understand the drivers and barriers in deployment and use of a smartphone app in a naturalistic setting and broad study population of people with and without multiple sclerosis (MS). The study utilised the Floodlight Open app: a ‘bring-your-own-device’ solution that remotely measures a user’s mood, cognition, hand motor function, and gait and postural stability via smartphone sensor-based tests requiring active user input (‘active tests’). Levels of mobility of study participants (‘life-space measurement’) were passively measured. Study data from these tests were made available via an open-access platform. Data from 1350 participants with self-declared MS and 1133 participants with self-declared non-MS from 17 countries across four continents were included in this report. Overall, MS participants provided active test data for a mean duration of 5.6 weeks or a mean duration of 19 non-consecutive days. This duration increased among MS participants who persisted beyond the first week to a mean of 10.3 weeks or 36.5 non-consecutive days. Passively collected life-space measurement data were generated by MS participants for a mean duration of 9.8 weeks or 50.6 non-consecutive days. This duration increased to 16.3 weeks/85.1 non-consecutive days among MS participants who persisted beyond the first week. Older age, self-declared MS disease status, and clinical supervision as part of concomitant clinical research were all significantly associated with higher persistence of the use of the Floodlight Open app. MS participants performed significantly worse than non-MS participants on four out of seven active tests. The findings from this multinational study inform future research to improve the dynamics of persistence of use of digital monitoring tools and further highlight challenges and opportunities in applying them to support MS clinical care.

## Introduction

Historically, multiple sclerosis (MS) was categorised as either relapsing or progressive, with relapses associated with focal inflammation and neurodegeneration, resulting in neurological progression that can be difficult to accurately quantify over time^1^. It is now understood that many patients who do not experience clinical relapses can still experience insidious disease progression throughout their disease course^2^, suggesting that the biological processes underpinning relapsing and progressive disease exist in patients across the entire MS disease continuum^3^. Thus, it is evident that there is a need for more frequent, sensitive, and accurate measures to evaluate the evolution of MS progression over time.

Conventional clinical assessment of MS relies on periodic in-clinic visits, typically every 6–12 months, or at the time of a relapse. Such infrequent observations can fail to comprehensively capture relapses or insidious deterioration that occur in between visits. In-clinic evaluations are further limited as they rely on patients’ recall of events and are not conducted in their natural environment.

Digital health technology tools such as smartphone sensor-based, remotely administered digital tests represent a promising new avenue to observe, with quantitative accuracy, disability progression or relapse activity more objectively and more frequently in the patient’s natural environment. Given the widespread availability of smartphone technology, these tests may be able to monitor disease course in a time- and cost-effective manner, and on a broad scale^4–6^. A German study in 2013 found that 95% of people with MS (PwMS) had access to a smartphone device^7^, thus demonstrating that smartphone assessment may be an accessible tool to evaluate MS in many settings.

Currently, several studies are investigating the use of digital health technology tools and smartphone apps in remote settings in MS^8–10^. The Floodlight technology uses smartphone-based digital tests to facilitate functional assessments in PwMS^5^. These self-administered tests assess key functional domains affected by MS, including cognition, hand motor function, and gait and postural stability. A 24-week, clinician-supervised, proof-of-concept study of the Floodlight technology demonstrated test–retest reliability (high intraclass correlations across the different assessments) and significant concordance with established clinical tests of references such as the Nine-Hole Peg Test, the Symbol Digit Modalities Test, the Timed 25-Foot Walk Test, the Expanded Disability Status Scale, and with MRI measures^10,11^. Additionally, the Floodlight technology showed high patient engagement and satisfaction over the 24-week study period^5^. However, further investigation into factors influencing the use of smartphone-based tests designed to monitor MS disease progression outside the rigors of clinic-based studies, where onboarding from a clinician may impact user engagement, is necessary as this is an important determinant of their real-world utility.

The Floodlight Open study aimed to explore in a broad, multinational study population the use of the Floodlight technology in a naturalistic setting, i.e. in the absence of predetermined external supervision or participant onboarding from a research team and/or healthcare professionals (HCPs). This study forms a part of the framework used to evaluate the suitability of the Floodlight technology for PwMS, further building on the findings described from the proof-of-concept study and constituting the first investigational deployment of Floodlight as a ‘bring-your-own-device’ (BYOD) solution. Here, we present the concept and methodology of the Floodlight Open study and describe data collected with the Floodlight Open app. Specifically, we evaluated the use of the Floodlight Open app, including the drivers and barriers in deployment and continued use, by analysing participant persistence; assessed differences in performance on the Floodlight Open tests between self-declared MS and non-MS participants; and evaluated the utility of applying objective quality control criteria that identify behaviours that suggest an unwillingness to complete the tests.

## Methods

### Study design and participants

Floodlight Open was a global, open-access, digital-only study in MS designed to understand the use of the Floodlight technology in a naturalistic setting and in a broad, multinational study population. Adult participants (age: ≥ 18 years) with and without self-declared MS from 17 participating countries across four continents (Supplementary Table S1) could enrol if they had a suitable smartphone with the operating system (OS) iOS (v.11.x or higher) or Android OS (v.7.x or higher) and provided e-consent. All participants downloaded the Floodlight Open app from their local app store onto their own smartphones. Through the app, they completed a set of smartphone-based patient-reported outcomes (PROs) and ‘active tests’ (i.e. requiring active user input) that assess mood, cognition, hand motor function, and gait and postural stability. Data on mobility level (life-space measurement) were collected passively. All active tests and life-space measurement leveraged the smartphone’s embedded sensors. As study enrolment and participation was entirely self-driven, no predetermined clinic visits or supervision and onboarding by clinicians, HCPs, or research teams were included in the study. Consequently, no clinician-reported outcomes were captured. Furthermore, awareness-raising initiatives around the study were not systematically and consistently deployed across countries during the recruitment.

The study protocol, informed consent forms, and relevant supporting information were reviewed and approved by the appointed central institutional review boards/ethics committees before the study was initiated in each participating country, as applicable, in accordance with each country’s regulatory requirements (Supplementary Table S2).

Smartphone data were collected for as long as the participants remained in the study as there was no predetermined study duration. Data were collected from 22 April 2018 (first participant enrolled) until 11 October 2021 (data cut-off date). At the time of data cut-off, Floodlight Open was still ongoing, but a gradual closure was planned in the subsequent months.

### Study workflow

**Figure 1:**
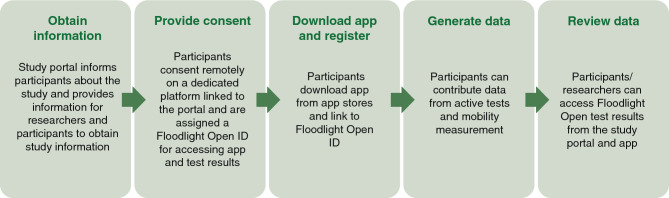
Overview of the Floodlight Open study workflow. Consent could be withdrawn at any time during the study, resulting in the termination of further data collection from the withdrawn participant. All data provided up to the withdrawal of consent were retained and used for study purposes. The Floodlight Open app was developed for iOS- and Android-based smartphones and made available for download in 17 countries (see Supplementary Table S1 for release dates in each country). *ID* identification, *iOS* iPhone operating system.

To support self-driven study enrolment and participation, a digital ecosystem was developed (Fig. 1), which comprised a web-based study portal to inform potential participants and researchers and to allow interrogation of pseudonymised study data, an e-consent platform, and the Floodlight Open app. After providing e-consent remotely on a secured, dedicated platform linked to the study portal, participants received an electronic token via email with which they could unlock the functionalities of the Floodlight Open app. Once the app was unlocked, they could complete registration, provide demographic and participant information (e.g. self-declared year of birth, sex, diagnosis of MS, weight, height, and country of residence), and start self-administering the Floodlight Open tests. Additionally, participants were able to access at any time information on the study, their e-consent, their generated data (including graphical representation of data), and terminate study participation, if desired.

### Floodlight Open app

#### PRO, active tests and life-space measurement

The Floodlight Open app is a BYOD solution that participants download onto their own smartphones. The concepts and technology of the smartphone sensor-based tests have been previously described^5,10^. The Floodlight Open tests included one PRO and several active tests that assessed mood (Daily Mood Questionnaire [DMQ]), cognition (Information Processing Speed [IPS] Test and IPS Digit–Digit Test), hand motor function (Pinching Test and Draw a Shape [DaS] Test), postural stability and gait (Static Balance Test [SBT], U-Turn Test [UTT], and Two-Minute Walk Test [2MWT]), and passively collected ‘life-space measurement’, which assessed the level of mobility of study participants^12^. The app prompted participants to take the PRO and active tests in a fixed sequence. This sequence started with the DMQ, which was followed by the IPS Test, IPS Digit–Digit Test, Pinching Test, DaS Test, SBT, and finally UTT. The only active test not included in this fixed sequence was the 2MWT as it required participants to walk straight for 2 min, something which may not always have been possible at the time the other active tests were being performed. The PRO and active tests could be completed up to once daily (apart from the IPS and IPS Digit–Digit tests, which could be performed up to once weekly), with an actual frequency that could be adjusted to individual preferences. The UTT, SBT, and 2MWT could be skipped for the day if participants determined the safety conditions for their execution, including environmental and physical factors, could not be met that day. The option to activate configurable reminders and notifications that reminded the participants to take these active tests was introduced a few months after the beginning of the study, at the time when, overall, only a small cohort of participants in the USA had been included and had started using the app.

An additional differentiating element from other analogous Floodlight app versions belonging to the same initial generation was that the Floodlight Open app passively measured mobility levels via the life-space measurement. This measure was defined as the distance between the two farthest Global Positioning System (GPS) coordinates detected during the day that were ≥ 500 m apart. The GPS coordinates were obfuscated and excluded from the public dataset to protect participants’ privacy.

Performance on the Floodlight Open tests were quantified through digital measures, which were computed from the raw smartphone sensor signals recorded during the execution of these tests (Supplementary Table S3). In this study, performance was computed for each completed test using the first version of the computing algorithms, except for the DaS Test and UTT, for which the latest algorithm was used to ensure optimal data comparability across different OS platforms.

Finally, participants were able to view their personal performance over time (Supplementary Fig. S1), a feature that was not previously available with the Floodlight technology.

#### Data management and availability

Demographic data and digital measures (i.e. smartphone-based PRO, active tests and passively collected life-space measurement data) were electronically collected and encrypted through the Floodlight Open app and stored on two specific, secure cloud databases that were controlled and maintained by the study initiator, F. Hoffmann-La Roche Ltd. The two databases were housed in the European Union and in the USA to comply with specific regulatory requirements on data storage and management.

To ensure confidentiality, all participants’ information collected after successful completion of the informed e-consent process and the download and activation of the Floodlight Open app were pseudonymised through association with a unique Floodlight Open participant identification (ID). Data collected while executing the Floodlight Open tests contained no personal identifiable information. Such information was only provided during the consent process (e.g. a valid email address), and these data were protected by securely storing this information separately from all other study data for the duration of the study in the e-consent provider’s record.

Pseudonymised Floodlight Open study data collected via the app were made publicly available through the Floodlight Open study dataset, which could be viewed and downloaded from the study portal. MS researchers thus had the opportunity to integrate the Floodlight Open app in their own research projects by appropriately obtaining consent from their patients to share their Floodlight Open study participant ID and leveraging individual datasets gathered in the open-access registry. Additionally, raw sensor data and associated metadata (e.g. OS version) were collected, transferred, and stored in a separate database whenever participants were connected to a WiFi network. This dataset was maintained by the study sponsor and is used to support ongoing efforts to further develop the Floodlight technology. Consequently, this dataset was not made publicly available.

### Data analysis

The analyses described below were exploratory and primarily descriptive. Statistical tests were conducted at the two-sided 5% significance level.

#### Persistence of use

Four different persistence of use definitions, or metrics, were used to assess persistence of participants with the Floodlight Open app. Participants were considered as being persistent for a given study week if they either: (i) completed at least any one test of the fixed sequence that week; (ii) completed at least one 2MWT that week; (iii) completed at least one full fixed test sequence that week (i.e. performed the whole fixed test sequence at least once in a week); or (iv) provided life-space measurement data for at least 1 day that week. If a participant completed the whole fixed test sequence on weeks 1, 3, and 6, for example, then this participant was considered persistent according to definition (iii) for 3 weeks.

Persistence of use was described for all evaluable participants and for those participants who persisted beyond the first week, who might show a distinctly different usage behaviour^8^. In the latter cohort, the proportion of participants who were considered persistent after 4, 8, 12, 24, and 52 weeks was also assessed. The impact of country on persistence was studied using descriptive statistics.

To better understand the drivers of persistent use, participants were grouped by demographic variables (e.g. MS vs. non-MS participants, female vs. male participants, participants using iOS vs. Android devices). Differences in these subgroups were assessed for statistical significance using the Mann–Whitney U test. Effect sizes of these differences (Cohen’s d)^13^ were also reported. To study the impact of age on persistence, participants were grouped into one of two subgroups: (i) those who were persistent for 5 weeks or more and (ii) those who were persistent for less than 5 weeks. Age differences between these two subgroups were also described by effect sizes (Cohen’s d) and assessed for statistical significance by the Mann–Whitney U test. Additionally, to better understand the impact of intermittent use of the app on persistence, the maximum duration of a break a participant took from using the Floodlight Open app before returning to using the app was correlated against their persistence with Spearman’s rank correlation. To ensure this correlation analysis used a sufficient number of data points, only participants who contributed to at least 10 test executions on at least one of the Floodlight Open tests were included.

#### Differences in test performance between MS and non-MS participants

Differences between MS and non-MS participants in performance on the Floodlight Open tests after adjustment for age and sex were assessed for statistical significance using multiple robust linear regression (Python package statsmodels v0.12.2)^14^. Cohen’s d effect sizes of these differences as well as of the impact of age and sex on test performance were reported. To exclude practice or learning effects^15^ that could influence results, this analysis was restricted to the first test run completed by each participant.

#### Impact of data quality on persistence

Alongside computing digital measures, the raw sensor data collected during test execution were used to identify single tests characterised by an unwillingness to complete the test (i.e. ‘play-to-quit’ behaviours). The criteria applied to identify such play-to-quit behaviours have been previously described^10^ and include: selecting a response independently from the shown symbol or digit on the IPS and IPS Digit–Digit tests, respectively; not interacting with the touch screen during the Pinching Test or DaS Test; or leaving the smartphone on a flat surface such as a table during the SBT, UTT, or 2MWT. Such criteria could be used as an objective assessment of the quality of the data collected in a remote, unsupervised, naturalistic setting.

To evaluate the impact of play-to-quit behaviours on our persistence findings, we assessed whether such behaviours were associated with persistence.

#### Data sources

The analysis on the persistence of use and the differences in test performance between MS and non-MS participants were conducted on the publicly available dataset. By comparison, the analysis of data quality (required to identify play-to-quit behaviours) leveraged the raw sensor data from a separate dataset.

## Results

### Consent, downloads, and participant demographics

The Floodlight Open study was conducted in 17 countries across four continents (Supplementary Table S1). As of 11 October 2021, 3 years and 6 months after the first country’s study activation (USA), the Floodlight Open app was downloaded 5180 times. A larger number of downloads was registered on iOS devices (n = 4208 [81.2%]) than on Android devices (n = 972 [18.8%]).

Of the 5108 participants who created a study account, 3976 (77.8%) provided full e-consent (143 [2.8%] participants withdrew e-consent while enrolled in the study) and 2519 (49.3%) provided data (generated digital measures) via the app. Thirty-six (1.4%) participants were excluded from the analyses as they recorded an age of < 18 years in violation with study inclusion criteria. This resulted in an evaluable study population of 2483 (48.6%) participants. Of these, 1099 (44.3%) participants were from the USA (Supplementary Table S1). Consistent with the global MS population^16^, the proportion of female participants was higher among MS participants (71.3%) than among non-MS participants (47.6%) (Table 1).

**Table 1:** Demographics of all evaluable participants.

Variable	Self-declared MS participants n = 1350	Self-declared non-MS participants n = 1133
Age (years), mean (SD)	45.2 (12.1)	39.5 (12.3)
Female, n (%)	962.0 (71.3)	539.0 (47.6)
Height (cm), mean (SD)	167.9 (18.8)	172.5 (15.8)
Weight (kg), mean (SD)	80.6 (26.1)	77.0 (21.6)
BMI (kg/m^2^), mean (SD)*	28.0 (9.0)	25.7 (9.4)

*BMI* body mass index, *MS* multiple sclerosis, *SD* standard deviation.

*Twenty-three participants (0.93% of all evaluable participants) reporting a height of ≤ 75 cm who were excluded as entries were deemed to be implausible.

### Floodlight Open app usage metrics

When all countries had initiated the study and full enrolment capacity potential was considered to have been attained (January 2020 to October 2021), the Floodlight Open app had a mean of 90 daily users, including 66 with MS, who provided PRO, active test or life-space measurement data. When considering PRO and active test data only, but not life-space measurement data, the app had a mean of 33 daily users, including 29 with MS. These 33 daily users generated on average 477 PRO/active test executions (428 test executions for users with MS). By comparison, a mean of 86 daily users, including 63 with MS, contributed life-space measurement data. Across all Floodlight Open tests, life-space measurement generated the largest volume of data points (Supplementary Tables S4–S7).

#### Drop-out rate from the fixed test sequence in participants with MS

**Figure 2:**
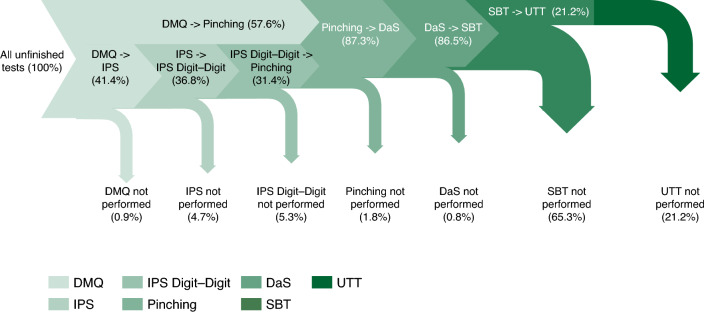
Funnel plot depicting the proportion of self-declared MS participants that progress from one active test (or PRO) to another active test, or drop out, during the fixed test sequence. Participants who never completed a full fixed test sequence were included. *DaS* Draw a Shape, *DMQ* Daily Mood Questionnaire, *IPS* Information Processing Speed, *MS* multiple sclerosis, *PRO* patient-reported outcome, *SBT* Static Balance Test, *UTT* U-Turn Test.

Participants took the DMQ and all active tests except the 2MWT in a fixed sequence. Over the entire study, the mean duration to execute the entire sequence was 5.3 min. Since the IPS and IPS Digit–Digit tests could only be taken weekly rather than daily, only a subset of the fixed test sequences included these cognitive tests. Among MS participants who did not complete any full test sequence (n = 284), 99.0% completed the DMQ, 41.4% completed IPS and IPS Digit–Digit tests, and 57.6% proceeded straight to the Pinching Test. The largest drop-out was observed with the SBT, where 65.3% of MS participants with incomplete test sequences opted not to take the test and dropped out from the fixed test sequence (Fig. 2).

### Persistence of use

#### Persistence in the evaluable study population

**Figure 3:**
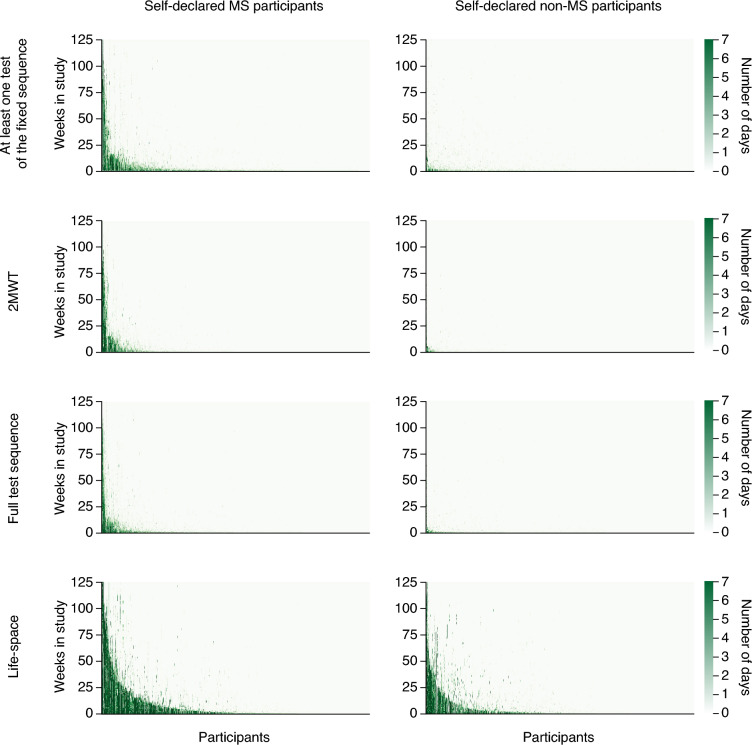
Persistence among participants. Persistence was defined as one of the following: at least any one test of the fixed sequence performed per week, at least one 2MWT performed per week, at least one full fixed test sequence performed per week, or having provided life-space measurement data in a given week. Participants are ordered along the x-axis starting with those with the highest persistence (self-declared MS, n = 1350; self-declared non-MS, n = 1133). The green shading indicates the exact number of days that the participant was adherent in the specific week. *2MWT* Two-Minute Walk Test, *MS* multiple sclerosis.

Persistence was measured according to one of four predefined persistence definitions. Of 2483 participants included in the evaluable study population, 2338 (94.2%) completed at least one test in the fixed test sequence, 991 (39.9%) completed at least one 2MWT, 1756 (70.7%) completed at least one full fixed test sequence, and 1953 (78.7%) provided at least 1 day of life-space measurement data. Across all persistence definitions (Fig. 3), a considerable proportion of participants (38%) ceased to participate after 1 week. Yet, there were 31 participants who interacted with the app (i.e. completed at least one test of the fixed test sequence per week) for more than 50 weeks, and five participants for over 100 weeks.

Compared with non-MS participants, MS participants exhibited markedly higher persistence across all persistence definitions (Table 2), with respect to persistent weeks on study and number of days of data provided. When considering the persistence definition of completing at least one test of the fixed test sequence per week, MS participants were persistent for a mean duration of 5.6 weeks, generating data for a mean of 19 non-consecutive days. Non-MS participants, by comparison, were persistent for a mean duration of 2.3 weeks, generating data for a mean of 4 non-consecutive days. When considering life-space measurements, MS participants were persistent for a mean duration of 9.8 weeks, generating data for a mean of 51 non-consecutive days and non-MS participants for a mean duration of 6.3 weeks and 28.5 non-consecutive days (Table 2).

**Table 2:** Persistence metrics by self-declared MS status for all evaluable participants and for participants who stayed in the study for more than 1 week.

Persistence definition	Parameter	Participants persisting beyond first week n = 1540 (mean)*		All participants N = 2483 (mean)	
		Self-declared MS	Self-declared non-MS	Self-declared MS	Self-declared non-MS
PRO and active tests					
At least one test of the fixed sequence performed per week	Weeks/days with data recorded	10.29/36.50^†^	4.28/7.67^‡^	5.59/18.98	2.30/3.93
At least one 2MWT performed per week	Weeks/days with data recorded	13.36/49.30^¶^	4.57/10.09^§^	3.59/12.71	0.60/1.15
At least one full fixed test sequence performed per week	Weeks/days with data recorded	9.52/30.51^||^	3.57/6.86^#^	3.70/11.22	1.13/1.88
Passive monitoring					
At least 1 day of life-space measurement data provided per week	Weeks/days with data recorded	16.33/85.12**	11.56/53.60^††^	9.84/50.65	6.29/28.53

*2MWT* Two-Minute Walk Test, *MS* multiple sclerosis, *PRO* patient-reported outcome.

*The mean weeks/days only includes participants with data for a given persistence definition.

^†^n = 672.

^‡^n = 467.

^¶^n = 338.

^§^n = 97.

^||^n = 468.

^#^n = 200.

**n = 793.

^††^n = 588.

**Figure 4:**
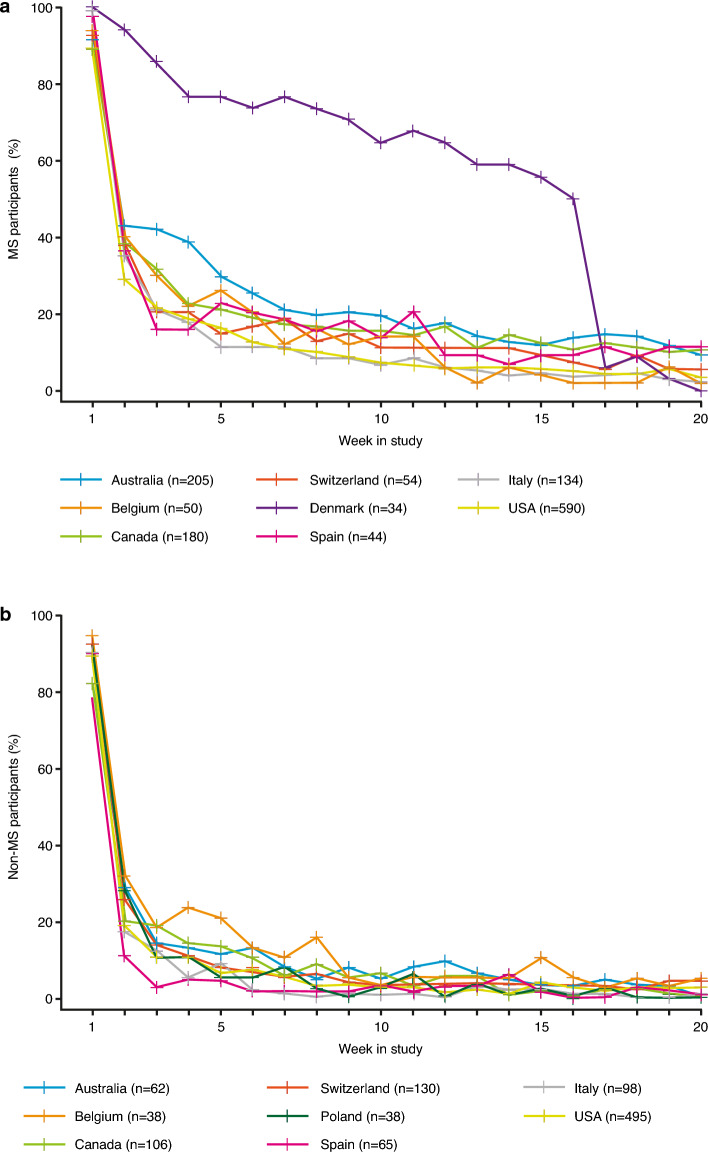
Weekly persistence among self-declared MS and non-MS participants by country in the first 20 weeks. Persistence of self-declared (**a**) MS and (**b**) non-MS participants per week (at least one test of the fixed sequence performed per week) across countries with at least 20 participants; numbers of participants shown are those who performed at least one test of the fixed sequence. *MS* multiple sclerosis.

Marked differences in weekly persistence (i.e. completing at least one test of the fixed sequence per week) were also observed between participant countries (Fig. 4). Notably, Danish participants, who were also part of a parallel 16-week MS care research study where they performed Floodlight Open tests as part of a physiotherapy protocol^17,18^, had higher persistence relative to other countries over the first 16 weeks in the study. Specifically, a mean of 71.7% of participants with MS from the Danish cohort were persistent during this timeframe compared with only 20.6% of participants with MS across all other countries. Self-declared non-MS participants’ weekly persistence across all countries followed the same persistence rate and drop-out trend as participants with self-declared MS, when excluding Danish participants from the self-declared MS cohort (Fig. 4).

#### Persistence beyond the first week of participation

Having acknowledged a substantial drop-out among participants during the first week (Figs. 3, 4), we assessed persistence among the 1540 participants (62% of the evaluable study population) who continued to interact with the app beyond the first week to focus on a population that may be more motivated to participate in the study. Among MS participants, mean persistence ranged from 9.5 weeks for completing at least one full fixed test sequence per week to 16.3 weeks for providing at least 1 day of life-space measurement data per week (Table 2).

To further characterise patterns of persistence, we evaluated persistence longitudinally among these MS participants. The metrics of all four persistence definitions consistently decreased over time, with passively collected life-space measurement data showing the highest persistence for the most time points (Table 3). Across definitions, 42.9–64.8% of participants were considered persistent for at least 4 weeks, which decreased to 3.6–8.3% after 52 weeks.

**Table 3:** Longitudinal persistence of self-declared MS participants.

Persistence definition	Proportion of self-declared MS participants persisting beyond 1 week who achieved reference threshold (%)*				
	> 4 weeks	> 8 weeks	> 12 weeks	> 24 weeks	> 52 weeks
At least one test of the fixed sequence performed per week^†^	47.5	28.9	20.8	8.6	4.2
At least one 2MWT performed per week^‡^	52.7	37.0	26.0	11.5	7.4
At least one full fixed test sequence performed per week^¶^	42.9	26.1	18.2	7.7	3.6
At least 1 day of life-space measurement data provided per week^§^	64.8	46.2	34.7	18.3	8.3

*2MWT* Two-Minute Walk Test, *MS* multiple sclerosis.

*When analyses were restricted to self-declared MS participants who persisted beyond 1 week and enrolled at least 52 weeks prior to data cut-off, the proportion of MS participants achieving the reference thresholds increased by 1.4 to 7.0% points across persistence definitions and timepoints.

^†^n = 672.

^‡^n = 338.

^¶^n = 468.

^§^n = 793.

#### Drivers of persistence in the evaluable study population

To identify drivers of persistent use, we evaluated how different demographic variables impacted persistence. Self-declared MS disease status was associated with higher persistence across all four definitions (all p < 0.001 vs. self-declared non-MS disease status). Furthermore, female sex was associated with significantly higher persistence on the 2MWT among all participants (p = 0.029).

The relationship between OS platform and persistence was also evaluated (Supplementary Table S8). iOS devices were associated with higher persistence compared with Android devices in terms of completing one full fixed test sequence per week in all participants (p = 0.011) and in MS participants (p = 0.008). iOS devices were also associated with higher persistence across all participants (p < 0.001) and in MS participants (p < 0.001) when considering life-space measurement data. However, significantly more participants (81.2%) used iOS smartphones.

More persistent participants (i.e. those persistent for 5 weeks or longer vs. for less than 5 weeks) were associated with older age in both MS participants (all p < 0.001) and non-MS participants (p = 0.015) (Supplementary Table S9). No other demographic variable, including body mass index, showed an association with persistence for any definition or population.

Interestingly, many participants demonstrated intermittent usage with breaks of weeks or months before returning to use the app (Fig. 3), which was also observed in prior studies^8^. Thus, taking a break from using the app was also assessed as a potential driver of persistent use. Such breaks were observed in 344 MS participants and 53 non-MS participants who had at least 10 test executions in one or more Floodlight Open test. Among these participants, longer breaks of at least 50 days were noted in 36.3% of MS participants and in 41.5% of non-MS participants. While correlations between the maximum break duration of a participant and the number of weeks they were persistent were generally low when considering participants irrespective of their MS status, they were higher for non-MS participants than for MS participants. The strongest correlations were observed between maximum break duration and higher persistence in terms of either completing at least one test of the fixed sequence per week (non-MS participants: Spearman’s rho = 0.670, p < 0.001; MS participants: Spearman’s rho = 0.262, p < 0.001) or providing life-space measurement data for at least 1 day per week (non-MS participants: Spearman’s rho = 0.522, p < 0.001; MS participants: Spearman’s rho = 0.392, p < 0.001) (Supplementary Table S10).

### Test performance

**Figure 5:**
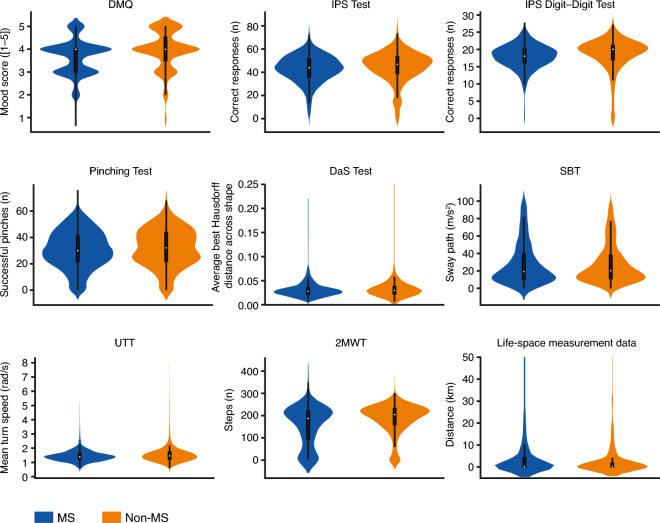
Distribution of test performance data for self-declared MS and non-MS participants. The distribution of performance data for each active test and life-space measurement is depicted with violin plots with low-degree smoothing applied. Note that for the DaS Test, the maximum average Hausdorff distance recorded for the non-MS population is 0.68. *2MWT* Two-Minute Walk Test, *DaS* Draw a Shape, *DMQ* Daily Mood Questionnaire, *IPS* Information Processing Speed, *MS* multiple sclerosis, *s* seconds, *SBT* Static Balance Test, *UTT* U-Turn Test.

The distribution of digital measures for each active test is presented in Fig. 5. These digital measures were associated with self-declared MS status, age, and sex. For example, not having MS was associated with better mood and better performance on the IPS, IPS Digit–Digit, and Pinching tests (all p < 0.001) as well as the UTT (p < 0.05; adjusted for age and sex) (Table 4).

**Table 4:** Differences in performance (between-group mean difference) between self-declared MS and non-MS participants at baseline, after adjusting for age and sex.

Test	Digital measure	Self-declared MS vs. non-MS between-group difference		
		RLR model coefficient (95% CI)	p-value	Effect size
PRO				
DMQ	Mood score (1–5)	0.358 (0.284, 0.432)	** < 0.001**	**− 0.031**
Active tests				
IPS Test	Correct response, n	3.181 (2.342, 4.020)	** < 0.001**	**− 0.054**
IPS Digit–Digit Test	Correct responses, n	0.918 (0.673, 1.164)	** < 0.001**	**− 0.083**
Pinching Test	Successful pinches, n	3.326 (2.108, 4.543)	** < 0.001**	**− 0.013**
DaS Test	Average best Hausdorff distance across shapes	− 0.001 (− 0.002, 0.000)	0.159	0.019
SBT	Sway path, m/s^2^	− 4.458 (− 9.000, 0.084)	0.054	0.130
UTT	Mean turn speed, rad/s	0.064 (0.024, 0.104)	**0.002**	**0.152**
2MWT	Steps, n	− 6.916 (− 14.301, 0.469)	0.066	− 0.050
Passive monitoring				
Life-space measurement data	Distance, km	1.261 (− 0.080, 2.602)	0.065	0.177

A positive value means that the non-MS participants had a higher value in the specific test feature (e.g. better mood, more IPS correct responses, more pinches, more accurate DaS results, more movement in the SBT, i.e. less balance, higher turning speed in the UTT, more steps in the 2MWT, and larger distance covered in life-space). A negative value means that MS participants had a higher value in the specific test feature.

*2MWT* Two-Minute Walk Test, *CI* confidence interval, *DaS* Draw a Shape, *DMQ* Daily Mood Questionnaire, *IPS* Information Processing Speed, *MS* multiple sclerosis, *PRO* patient-reported outcome, *RLR* robust linear model, *s* seconds, *SBT* Static Balance Test, *UTT* U-Turn Test.

Bold indicates statistical significance.

In all study participants (MS and non-MS), older participants performed better on the DMQ and SBT, but worse on the IPS Test, IPS Digit–Digit Test, Pinching Test and UTT (all p ≤ 0.001; Supplementary Table S11). These observations held true in both non-MS and MS populations, with minor differences. Among MS participants, older participants also performed significantly worse on the 2MWT (p = 0.009) while the association with DMQ (p = 0.013) was marginal (Supplementary Table S11). Furthermore, older age showed no association with performance of MS participants on the SBT (p = 0.444). Among non-MS participants, the associations with older age and better performance on the DMQ (p = 0.012), SBT (p = 0.015), and 2MWT (p < 0.001) were marginal. There was no significant association of age with life-space in any of the study populations (Supplementary Table S11).

Sex was also significantly associated with several Floodlight Open tests. Among MS participants, male participants performed worse on the IPS Test (p = 0.033) and SBT (p = 0.043), better on the Pinching Test (p = 0.002), and marginally better on the DaS Test (p = 0.018) than female participants (Supplementary Table S12). Among non-MS participants, male participants walked fewer steps on the 2MWT than female participants (p < 0.05; Supplementary Table S12).

### Impact of data quality on persistence

Quality control data were available for a subset of 1938 participants. Demographics between these participants and all evaluable participants were comparable (Supplementary Table S13). The proportion of test executions that did not show signs of play-to-quit behaviours was high across all active tests, ranging between 86.3 and 99.9%. Compared with non-MS participants, MS participants were less likely to show play-to-quit behaviours (Table 5).

**Table 5:** Proportion of active tests that were considered free of play-to-quit behaviours.

Functional domain	Test	Sensors used	Digital measure	Number of test performed without play-to-quit behaviour, % (n/N)			Quality control criterion
				Self-declared MS participants	Self-declared non-MS participants	All participants	
Cognition	IPS Test	Touchscreen	Correct response, n	99.7% (4073/4086)	86.3% (794/920)	97.2% (4867/5006)	Response selected independently of symbol to be matched
Cognition	IPS Digit–Digit Test	Touchscreen	Correct responses, n	99.7% (3950/3962)	88.5% (710/802)	97.8% (4660/4764)	Response selected independently of symbol to be matched
Hand motor function	Pinching Test	Touchscreen	Successful pinches, n	99.9% (22,521/22,537)	99.3% (3212/3235)	99.8% (25,733/25,772)	No gestures recorded by touchscreen (no screen interaction)
Hand motor function	DaS Test	Touchscreen	Average best Hausdorff distance across shapes	87.5% (22,053/25,198)	61.2% (3659/5980)	82.5% (25,712/31,178)	No touch events for any of the six shapes
Gait and balance	SBT	Accelerometer	Sway path, m/s^2^	96.2% (18,951/19,693)	89.1% (2272/2550)	95.4% (21,223/22,243)	Phone is kept on the table
Gait and balance	UTT	Accelerometer, gyroscope	Mean turn speed, rad/s	98.2% (17,528/17,846)	96.6% (2060/2133)	98.0% (19,588/19,979)	Phone is kept on the table
Gait and balance	2MWT	Accelerometer	Steps, n	99.6% (16,203/16,261)	97.9% (1193/1219)	99.5% (17,396/17,480)	Phone is kept on the table

*2MWT* Two-Minute Walk Test, *DaS* Draw a Shape, *IPS* Information Processing Speed, *MS* multiple sclerosis, *s* seconds, *SBT* Static Balance Test, *UTT* U-Turn Test.

All 100% of test executions by the 678 participants who completed only one test were free of play-to-quit behaviours. This proportion reduced slightly to 97.6% (SD: 6.7%) in participants who completed more than one test. The proportion of tests executed without play-to-quit behaviour showed no positive correlation with the number of weeks a participant was considered persistent according to any of the four definitions (Spearman’s rho: − 0.322 through – 0.225 for all evaluable participants; Spearman’s rho: − 0.215 through 0.085 for all participants persistent beyond the first week). Thus, an increase of play-to-quit behaviour was not found to be a predictor of decreased persistence with the Floodlight Open app.

## Discussion

The Floodlight Open study demonstrates the use of the Floodlight Open app in a large, multinational study population and the utility of an open-access, BYOD solution for medical research in MS. This study identified several drivers and barriers to the deployment and use of such a tool (Panel) and builds on prior smaller studies that have highlighted the feasibility and challenges of using remote, digital tools for MS clinical care^8,19,20^.

**Table:** 

Panel: Drivers and barriers in deployment and use of the Floodlight Open app in a remote, naturalistic setting
**Drivers** • Higher persistence was associated with: o Clinical referral o Older age o Self-declared MS status **Deployment barriers affecting recruitment** • Differences in local study evaluation and approval processes, which led to a fragmented study start-up worldwide • Lack of trustworthy human–human interactions during onboarding • Complexities related to developing and deploying both an iOS and Android version in several countries • Multiple platforms used for registration, e-consenting, and app activation processes • No systematic and consistent use of study awareness initiatives across countries **Barriers impacting adoption and continued use** • The mode of test administration, i.e. administering the active tests in a fixed sequence vs. independently of each other, impacting which tests are performed, which tests are skipped, and how often the tests are performed • Lack of motivational incentives provided through ongoing supervision by a healthcare professional or clinical researcher (because of the fully digital operational research environment)

In Floodlight Open, self-declared MS participants were considerably more persistent with using the Floodlight Open app than self-declared non-MS participants. Persistence of and adherence to smartphone-based tests have been assessed in previous studies in people with neurological disorders, including in people with MS^4,8^. Persistence trends observed in Floodlight Open, where participants with self-declared MS interacted with the Floodlight Open app at least once weekly for a mean of 5.6 weeks (19 non-consecutive days) and show a gradual decline in use over time, were in line with previously reported digital studies involving different populations and diseases^8,21^. Prior studies have shown that 25% of users abandon mobile apps after the first interaction^22^, and that 53% of users uninstall mobile apps within 30 days after download^23^, highlighting difficulties with persistent use of apps in the general population and making a case to identify and evaluate participant characteristics and factors that may increase persistence and adherence.

Our analyses revealed that persistent use up to 2–4 months is possible when focusing on the 62% of Floodlight Open’s participants who persisted beyond the first week. Compared with all evaluable participants, these participants demonstrated an increase in the mean duration of persistence by two to fourfold across each of the four persistence definitions (Table 2). After 4 weeks, 42.9–64.8% of these participants were considered persistent, with some persistent for as long as 50–100 weeks. Similar to previously reported findings, longer persistent use was also associated with self-declared MS status and older age^8^. Additionally, we observed that female sex, iOS platform, and taking breaks from using the Floodlight Open app were associated with some of the persistence metrics.

Although the participation in the study and usage of the app were not necessarily associated with benefits in clinical care, a concomitant investigator-supervised research study in Denmark likely provided motivational conditions that explains the markedly higher persistence observed in Danish participants^18^. Prior studies have also demonstrated that HCP involvement can be a driver of persistence^5,8^. For instance, HCP input and median study retention in an MS-focused digital health study were higher in clinically referred vs. self-referred participants^8^. Our findings, together with prior reports, underscore the point that patients are willing to spend time on remote monitoring tools if the app is used as part of a study or a treatment monitoring plan by a clinical team, providing insight into factors that may increase persistence for future digital efforts.

We observed two notable trends in persistence patterns. First, many participants showed breaks of weeks to months between interactions with the app. One hypothetical explanation for those participants with self-declared MS could be that they are returning to app usage due to a motivational prompt—possibilities include new concerning symptoms or a pending visit with their clinician. Second, some participants started interacting with the app by providing PRO and active test data but quit before completing a full fixed test sequence. When interrupted fixed test sequences by MS participants who never completed a full fixed test sequence were evaluated in detail, it became apparent that 86.5% terminated the test sequence during gait or postural stability tests (which require the participant to stand or walk); 65.3% were terminated before taking the balance test (SBT) and 21.2% ahead of the walking test (UTT). The reasons underlying these observations are variable, and likely include limitations related to MS, reduced preference/motivation, or reduced convenience. In many situations, standing or walking as part of an active test may be more difficult to perform, perhaps due to balance or walking concerns, or because the assessment is inconvenient and requires a substantial activity change, or disrupts current activities (e.g. performing the test during a commute, in a public area, or when resting). In support of the latter point, some MS participants performed the 2MWT, which was not included in the fixed sequence of PROs and active tests, persistently, despite some previously reported dissatisfaction with this test^5^. This observation suggests that there may be benefits in performing different tests independently from one another at a time that is convenient for the user, rather than performing them in a fixed sequence. Further exploration of these observations will provide practical insights into app-related factors underlying persistence and adherence, which will be useful to inform the development of future MS-related apps.

We also observed two notable trends in life-space measurements in Floodlight Open. First, when enabled, passively collected life-space measurements generated more data than all other data sources. Second, persistence was highest for life-space measurements. Our observations suggest that passively collected data may be key to generating longer-term datasets with minimal burden to study participants. This is in line with findings previously reported by independent researchers that passively collected measurements are preferable^24^. However, factors limiting the acceptance of passively collected data including issues with battery consumption and privacy concerns need to be addressed to facilitate this type of data collection.

Importantly, most of the Floodlight Open tests were able to differentiate between participants with self-declared MS and non-MS in a naturalistic setting, which is aligned with previous findings obtained from the Floodlight Proof-of-Concept study, where participants were supervised by clinicians^10^. In Floodlight Open, MS participants generally performed on average worse than non-MS participants. There were differential effects of age and sex on test performance in self-declared MS in comparison with non-MS participants: age impacted test performance in both MS and non-MS participants, whereas sex was only associated with performance on the 2MWT in non-MS participants. The lower average height in female vs. male participants may have resulted in smaller steps with higher cadence, resulting in a higher step count^25^.

To assess the quality of the collected data, we applied quality control criteria to the raw sensor data. Encouragingly, compared with the proof-of-concept study^10^, we observed similar proportions of test executions that showed no signs of play-to-quit behaviour. The small proportion of test executions characterised by such behaviours suggest that they had a negligible impact on our findings. Future analyses on participants showing no signs of play-to-quit behaviours could create a path to study effective engagement with remote digital assessments in a naturalistic setting. Additionally, these quality control criteria can also be used to remove test executions deemed invalid prior to performing statistical analyses^10^.

Independent qualitative research previously reported additional drivers and barriers of persistent use of the Floodlight Open app^24,26^. The use of reminders and notifications (note that most participants had this option available)^26^, a willingness to help clinical research^26^, and a perceived return on effort^24^ were identified as potential drivers. Furthermore, making test scores available to the participants may instil in some a competitive behaviour with oneself, thereby leading to a more persistent use of the app^24^. Making these test scores available may, however, also remind participants of their condition^24^. Furthermore, an essential and unchanging design and lack of gamification could potentially lead to boredom when performing repetitive tasks such as when regularly and frequently taking the Floodlight Open tests^24^. Current efforts are investigating the impact of enhancing the design and level of gamification of the Floodlight technology.

There are a number of limitations to our study. While the entirely remote and naturalistic setting of the Floodlight Open study enabled easy access for study participants, self-declared MS status and demographic data could not be verified (Supplementary Table S14) and may have included erroneous values as well as outliers. However, the impact of outliers on our observations is negligible as visible outliers occurred in only a very small proportion of data points. For example, only one (0.16%) participant reported an age > 100 years and 23 (0.93%) participants a height of < 75 cm. Moreover, despite not being able to verify self-reported clinical measures, we observed expected differences in most tests in MS vs. non-MS participants, supporting that most self-reported measures were correct. Second, the non-MS comparison group did not necessarily represent a healthy control group as the group could have included participants potentially suffering from other health conditions that may have affected test performance. In addition, there were no provisions in place to ensure participants performed the tests exactly as instructed by the app, which may have impacted test results. Nonetheless, our observations reflect those collected in a voluntary, naturalistic setting, and we demonstrated that objective quality control criteria can be applied to identify test executions characterised by an unwillingness to complete the test. Finally, we did not collect qualitative information about the perceived burden of different tests or on participant motivation to engage and continue to participate in the study, which are aspects that would be important to explore in future studies.

It is notable that in Floodlight Open 22.2% of participants downloaded the app and created a study account but did not complete the consenting process, and thus were ultimately not able to use the app. The underlying reasons are not clear, but we speculate may be multifactorial. Possible factors include participants wanting to ‘sample’ the app without participating in the research, discomfort with providing consent for research, or technological difficulties in completing the e-consent process and activating the app (which involved multiple platforms, including the e-consent platform, emails, the local app store, and the Floodlight Open app itself). Such scenarios are exacerbated by the absence of study investigators and human–human interactions to assist with the process and facilitate participation. The latter hypothesis is also supported by the high drop-out rate between account creation, completion of the e-consent, and first use of the app compared with the high persistence observed in other studies using Floodlight apps in which informed consent occurred in a supervised setting^5^. These findings highlight the need for a smooth, user-friendly onboarding process to facilitate persistence throughout the study.

Recruitment for Floodlight Open was influenced by a number of factors, including regulatory, technological, and methodological-/design-related factors. The breadth of geographical rollout resulted in a fragmented study start-up worldwide due to both a stepwise approach in activating the study in the different countries (i.e. a small number of countries activated per time period) and different local study evaluation and approval processes. Additionally, the complexity of app development resulted in considerably different timings of the availability of the iOS and Android platforms (the latter made available after iOS; see Supplementary Table S1). These and other factors likely contributed to observing a larger proportion of US participants in the study population due to both a longer recruitment period and a far higher iOS market penetration in the USA compared with other countries^27^. Conversely, the later availability of the Android app likely negatively affected recruitment, especially in the 10 countries that initiated participation in Floodlight Open most recently, where Android market penetration is higher.

Despite the challenges of local study evaluation and approval processes, the broad geographical rollout of Floodlight Open offered the opportunity to bring to the attention of local research approval bodies the concept of a fully remote research study, which enables digital data collection and sharing. Discussions with regulatory bodies helped achieve alignment and a common understanding of multiple aspects (regulatory, digital data security and privacy, methodology, and operational management) of digital health research. The most frequently debated aspects with local approving bodies during the start-up phase were items around the e-consent process, data handling and storage, provisions for data security, and clarifications around the intended purpose of the app. In some instances, localised versions of user-facing information across the different digital platforms were required to comply with local regulations (e.g. GDPR-compliant terms in the EU). These discussions were particularly frequent and interesting outside of the USA, where familiarity with such studies was still limited^28^ and under different regulatory frameworks. The hope is that such discussions with local regulatory bodies will facilitate the approval for future MS studies, which will inevitably become an increasing part of various types of research in the healthcare field and beyond in the years to come.

One notable aspect of Floodlight Open is that the infrastructure and data generated in the study were made easily usable and openly available to the research community for further investigation. At the time of this report, several researchers have already leveraged the Floodlight Open infrastructure and dataset, demonstrating clear interest in such datasets from the scientific community^12,18,29–31^. Furthermore, multiple institutions across several countries used the Floodlight Open study app in their own research, and others have utilised our data in their reports^12,18,30,31^. Such frameworks are already becoming an important component of collaborative research and information dissemination^19^, and have great potential to improve the impact of studies and make valuable progress in science.

This study provides several key learnings for the successful deployment of a remote digital health technology tool such as the Floodlight technology. One learning is that optimising the perceived value on effort by the patient user is critical for the continued use of the tool. This can be achieved by linking use of the tool with clinical care, or by associating its use with a supervised clinical research study. Both of these scenarios introduce a tangible benefit and motivational driver for users other than their willingness to contribute to advancing knowledge and science. Another learning is that HCPs or clinical researchers play a key role in the adoption of a digital health technology tool. Not only can they recommend the tool to the patient, but they can also help with the onboarding. Furthermore, reviewing the test scores during consultations can be an additional motivational incentive for patients to use the tool. Finally, the use of more effective user experience design features^32^ can help overcome technical barriers associated with the tool, help remind the users to adhere to the test schedule, and provide further motivational incentive.

## Conclusion

The Floodlight Open study evaluated the use of the Floodlight Open app in a large, multinational study population. The study’s architecture confirmed the feasibility of using a BYOD tool to assess performance across various neurological modalities, including cognition, hand motor function, and gait and postural stability of participants with self-declared MS; and provided valuable information on the challenges associated with achieving engagement and persistence over time. Such findings inform future development and evolution of digital health technologies in MS clinical care. Persistence outside the context of a supervised trial continues to be one of the most important hurdles in the adoption of remote digital health technology tools and finding effective strategies to improve the long-term engagement of people with MS remains an important and open challenge. However, given the chronic nature of MS, it is likely that event-driven and periodic patterns of use or less stringent frequencies of use can still offer insights into changing trends in physical and cognitive performance, representing interesting areas for further exploration. Future work could include further exploration of the user experience to increase persistence, further refinement and validation of specific tests to use as disease-monitoring tools in clinical care, and the integration of the app in clinical studies and MS clinical care.

### Supplementary Information

Supplementary Information.

## Data Availability

The Floodlight Open feature dataset was publicly available on the study web portal (www.floodlightopen.com) for the duration of the study. Up-to-date details on Roche’s Global Policy on Sharing of Clinical Study Information and how to request access to related study documents and to the dataset after decommissioning of the study web portal can be found here: https://www.roche.com/innovation/process/clinical-trials/data-sharing/. Anonymised records for individual patients across more than one data source external to Roche cannot, and should not, be linked due to a potential increase in risk of patient re-identification.
