# Supplementary material for: Use of smartphone-based remote assessments of multiple sclerosis in Floodlight Open, a global, prospective, open-access study
Source: Sci Rep. 2024 Jan 2;14:122. doi: 10.1038/s41598-023-49299-4 (PMC10762023; doi:10.1038/s41598-023-49299-4)
Supplement: Supplementary file 1 — Supplementary Information. [file 41598_2023_49299_MOESM1_ESM.docx]

**Supplementary Online Content**

**Use of smartphone-based remote assessments of multiple sclerosis in Floodlight Open, a global, prospective, open-access study**

Jiwon Oh^1^, Luca Capezzuto^2^, Lito Kriara^2^, Jens Schjodt-Eriksen^2^, Johan van Beek^2*^, Corrado Bernasconi^2^, Xavier Montalban^3^, Helmut Butzkueven^4^, Ludwig Kappos^5^, Gavin Giovannoni^6^, Riley Bove^7^, Laura Julian^8^, Mike Baker^2^, Christian Gossens^2^ & Michael Lindemann^2^

^1^Division of Neurology, St. Michael’s Hospital, University of Toronto, Toronto, ON, Canada.
^2^F. Hoffmann-La Roche Ltd., Basel, Switzerland. ^3^Department of Neurology–Neuroimmunology, Centre d’Esclerosi Múltiple de Catalunya (Cemcat), Hospital Universitari Vall d’Hebron, Barcelona, Spain. ^4^Department of Neuroscience, Central Clinical School, Monash University, Melbourne, Australia. ^5^Research Center Clinical Neuroimmunology and Neuroscience Basel (RC2NB), University Hospital Basel, University of Basel, Basel, Switzerland. ^6^Queen Mary University of London, London, UK. ^7^UCSF Weill Institute for Neurosciences, Department of Neurology, University of California San Francisco, San Francisco, CA, USA. ^8^Genentech, Inc., South San Francisco, CA, USA.

*At the time of writing Johan van Beek was an employee of F. Hoffmann-La Roche Ltd.; his current affiliation is Biogen Digital Health International GmbH, Baar, Switzerland.

Corresponding author:

Dr Luca Capezzuto

email: [luca.capezzuto@roche.com](mailto:luca.capezzuto@roche.com)

Tel.: +41 61 687 89 63

**Supplementary Table S1**. Demographics per country.

| **Country** | **Study activation**  **(iOS app release)** | **Android app release** | **Self-declared MS participants**  **n (%)**  **N=1,350** | **Self-declared non-MS**  **participants**  **n (%)**  **N=1,133** | **All**  **n (%)**  **N=2,483** |
| --- | --- | --- | --- | --- | --- |
| USA | Apr-2018 | Jul-2019 | 596 (44.1) | 503 (44.4) | 1,099 (44.3) |
| Canada | Sep-2018 | Jul-2019 | 181 (13.4) | 113 (10.0) | 294 (11.8) |
| Australia | Dec-2019 | Jun-2020 | 205 (15.2) | 63 (5.6) | 268 (10.8) |
| Italy | Jun-2019 | Oct-2019 | 134 (9.9) | 100 (8.8) | 234 (9.4) |
| Switzerland | Apr-2019 | Oct-2019 | 56 (4.1) | 141 (12.4) | 197 (7.9) |
| Spain | Jun-2019 | Oct-2019 | 44 (3.3) | 66 (5.8) | 110 (4.4) |
| Belgium | Dec-2019 | Jun-2020 | 50 (3.7) | 38 (3.4) | 88 (3.5) |
| Poland | Dec-2019 | Jun-2020 | 15 (1.1) | 38 (3.4) | 53 (2.1) |
| Denmark | Apr-2019 | Oct-2019 | 34 (2.5) | 17 (1.5) | 51 (2.1) |
| Brazil | Dec-2019 | Jun-2020 | 11 (0.8) | 19 (1.7) | 30 (1.2) |
| The Netherlands | May-2020 | Sep-2020 | 13 (1.0) | 8 (0.7) | 21 (0.8) |
| Finland | Dec-2019 | Jun-2020 | 1 (0.1) | 14 (1.2) | 15 (0.6) |
| Czech Republic | Jun-2019 | Oct-2019 | 5 (0.4) | 9 (0.8) | 14 (0.6) |
| Mexico | May-2020 | NA | 3 (0.2) | 3 (0.2) | 6 (0.2) |
| Estonia | Dec-2019 | Jun-2020 | 1 (0.1) | 1 (0.1) | 2 (0.08) |
| Sweden | May-2020 | NA | 1 (0.1) | 0 (0.0) | 1 (0.04) |
| Norway | Dec-2019 | Jun-2020 | 0 (0.0) | 0 (0.0) | 0 (0.0) |

iOS, iPhone Operating System; MS, multiple sclerosis; NA, not applicable.

**Supplementary Table S2**. Institutional review boards/ethics committees per country.

| **Country** | **Institutional review board/ethics committee** |
| --- | --- |
| Australia | Monash University Human Research Ethics Committee, Melbourne, Australia |
| Belgium | Commissie Medische Ethiek Imeldaziekenhuis Bonheiden, Bonheiden, Belgium |
| Canada | Western Institutional Review Board, Puyallup, WA, USA |
| Czech Republic | Etická komise Všeobecné fakultní nemocnice v Praze, Prague, Czech Republic |
| Estonia | Tallinna Meditsiiniuuringute Eetikakomitee, Tallinn, Estonia |
| Italy | Comitato di Etica dell’ Ospedale San Raffaele di Milano, Milan, Italy |
| Mexico | Comité de Ética en Investigación y Comité de Investigación de la Clínica Bajío CLINBA, S.C., Guanajuato, Mexico |
| Netherlands | De Adviescommissie nWMO Martini Ziekenhuis Groningen, Groningen, Netherlands |
| Spain | Comité de Ética de la Investigación con medicamentos (CEIm) del Parc Taulí de Sabadell, Barcelona, Spain |
| Sweden | Etikprövningsmyndigheten Sverige, Uppsala, Sweden |
| Switzerland | Ethikkommission Nordwest- und Zentralschweiz EKNZ, Basel, Switzerland |
| USA | Western Institutional Review Board, Puyallup, WA, USA |

In Brazil, Denmark, Finland, Norway and Poland an EC/IRB approval was not required for this research study type.

**Supplementary Table S3.**Description of smartphone-based PRO and active tests.

| **Domain** | **Test** | **Concept** | **Test description** | **Digital measure** | **Function concept assessed by the digital measure** |
| --- | --- | --- | --- | --- | --- |
| Mood | DMQ**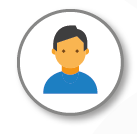** | Assess participant’s perceived overall mood. | Participants are asked to select one of five scaled answers that best describes their mood at the time of the test: excellent, good, fine, poor, or very poor. | - Mood score | Overall mood |
| Cognition | 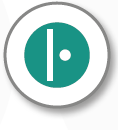IPS Test | Assess neurocognitive functions that underlie a visual iterative substitution task, including sustained attention, visual scanning, and recent memory. | Participants are presented with symbols that each represent the numbers 1–9. Over a period of 90 seconds, a series of symbols is presented, and the participant must select the correct corresponding number as fast as they can. | - The number of correct symbols matched - Average response time | Ability to recognise and match symbols and the speed of recognition |
|  | 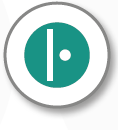IPS Digit–Digit Test | Following the IPS substitution task, participants must carry out a digit/digit matching exercise. The digits are presented in the same sequential order as the symbols in the prior substitution task. This task measures the visuomotor component of the matching task. During the IPS Test, the reaction time is the result of several mechanisms, including the primary focus (i.e. the Information Processing Speed). The IPS Digit–Digit Test is administered after the IPS and measures the other processes excluding the information processing speed so the contribution of the information processing mechanism can be isolated. | Participants are presented with a number (between 1–9) for a period of 15 seconds and are required to press the same number on the screen as fast as they can. | - The number of correct digits entered - Average response time | Ability to recognise digits and speed and accuracy of response to digit identification |
| Hand motor function | 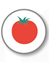Pinching Test | Assess fine distal motor manipulation (gripping and grasping) and control by evaluating accuracy of pinch closed finger movement. The test is considered to cover the following aspects of impaired hand motor function: impaired gripping/grasping function, muscle weakness, and impaired hand–eye coordination. | Participants are presented onscreen with a picture of a tomato. Using their thumb and index finger, participants have 30 seconds to squeeze as many tomatoes as possible. | - Number of successful pinches, n: number of tomatoes successfully pinched, or squeezed, within 30 seconds - Hand used during the test as instructed in the app for each test | Ability to synchronously perform fine distal upper extremity movement  Ability to perform fast and accurate finger opposition movement |
|  | Draw a Shape Test**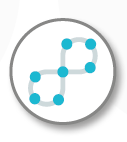** | Assess fine finger control and stroke sequencing. The test is considered to cover the following specific aspects of impaired hand motor function: tremor and spasticity and impaired hand–eye coordination. | At the beginning of the test a shape connected by dots appears on the screen. Starting at the largest dot, participants are required to follow the path of the shape with their index finger as quickly and as accurately as possible.  Once a shape is completed a new shape appears. | - Number of shapes drawn correctly - Best Hausdorff distance (distance of line drawn from the line displayed) per shape - Average best Hausdorff distance across all shapes - Hand used during the test as instructed in the app for each test | Accuracy of fine distal upper extremity movement  Speed and accuracy of fine distal upper extremity movement |
| Gait and postural stability | SBT 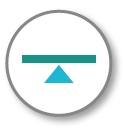 | Assess a participant’s static balance function while standing unsupported. | With the device in a front or back pocket, and after an initial vibration from the device, participants are instructed to stand upright with arms down and maintain balance until the device vibrates a second time.  Participants may use a walking aid if required for standing safely. | - Sway path, m/s^2^:   sum of the accelerometer signals in the x-, y-, and z-axis | Ability to maintain stable orthostatic posture |
|  | 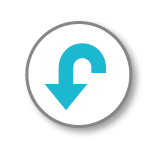UTT | Assess difficulties or unusual patterns in performing U-turns while walking a short distance at a comfortable pace. | With the device in a front or back pocket, participants are instructed to walk between two points positioned 4 metres apart and complete at least five turns in 60 seconds.  A vibration from the device signals the participant can begin walking until a second vibration 60 seconds later marks the end of the test.  Participants do not need to complete the test if they cannot walk safely, and they may use a walking aid if required for walking safely. | - Turn speed, rad/s:   angular velocity while performing U-turns   - Number of turns | Ability to turn while walking |
|  | 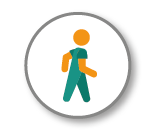2MWT | The aim of the 2MWT is to assess difficulties, fatigability, and unusual patterns in long-distance walking by capturing gait features. | With the device in a front or back pocket, participants are instructed to walk as fast and straight as possible, as long as it is safe to do so.  A vibration from the device signals the participant can start walking. A second vibration 2 minutes later marks the end of the test.  Participants do not need to complete the test if they cannot walk safely, and they may use a walking aid if required for walking safely. | - Number of steps | Pace maintained during regular walking in a defined time |

2MWT, Two-Minute Walk Test; DMQ, Daily Mood Questionnaire; IPS, Information Processing Speed; PRO, patient-reported outcome; s, seconds; SBT, Static Balance Test;
UTT, U-Turn Test.

**Supplementary Table S4.** Total number of tests executed across all participants for each smartphone-based test.

| **Test** | **Self-declared MS participants** | **Self-declared non-MS participants** | **All participants** |
| --- | --- | --- | --- |
| **PRO** | | | |
| **DMQ** | 25,718 | 4,459 | 30,177 |
| **Active tests** | | | |
| **IPS Test** | 6,836 | 2,059 | 8,895 |
| **IPS Digit–Digit Test** | 6,786 | 2,030 | 8,816 |
| **Pinching Test** | 24,930 | 3,712 | 28,642 |
| **DaS Test** | 24,768 | 3,542 | 28,310 |
| **SBT** | 20,754 | 2,837 | 23,591 |
| **UTT** | 18,761 | 2,371 | 21,132 |
| **2MWT** | 17,202 | 1,315 | 18,517 |
| **Passive monitoring** | | | |
| **Life-space measurement** | 68,761 | 32,428 | 101,189 |

PRO and active assessments can be executed up to a daily frequency except the IPS and IPS Digit–Digit, which can be executed up to a weekly frequency. Life-space measurement data are collected daily. 2MWT, Two-Minute Walk Test; DaS, Draw a Shape; DMQ, Daily Mood Questionnaire; IPS, Information Processing Speed; MS, multiple sclerosis; PRO, patient-reported outcome; SBT, Static Balance Test; UTT, U-Turn Test.

**Supplementary Table S5.** Total number of tests executed, number of participants, tests executed per participant, and range of data generated across active tests and life-space for all evaluable participants.

| **Test** | **Number of tests executed** | **n** | **Number of tests executed per participant** | **Quartiles of number of tests executed** | | | | |
| --- | --- | --- | --- | --- | --- | --- | --- | --- |
|  |  |  |  | **Min.** | **25%** | **50%** | **75%** | **Max.** |
| **DMQ** | 30,177 | 2,350 | 2 | 1 | 3 | 4 | 4 | 5 |
| **IPS Test** | 8,895 | 2,226 | 1 | 0 | 38 | 45 | 52 | 81 |
| **IPS Digit–Digit Test** | 8,816 | 2,218 | 1 | 0 | 16 | 19 | 21 | 27 |
| **Pinching Test** | 28,642 | 2,193 | 2 | 0 | 22 | 31 | 42.5 | 69 |
| **DaS Test** | 28,310 | 1,895 | 2 | 0.007 | 0.022 | 0.029 | 0.036 | 0.684 |
| **SBT** | 23,591 | 2,018 | 2 | 0 | 10.750 | 19.62 | 37.44 | 99.820 |
| **UTT** | 21,132 | 1,563 | 2 | 0.471 | 1.192 | 1.401 | 1.632 | 7.598 |
| **2MWT** | 18,517 | 997 | 2 | 0 | 148.5 | 202.5 | 228 | 382 |
| **Life-space measurement** | 101,189 | 1,960 | 12 | 0 | 0 | 0 | 3.1 | 165.25 |

Median used per participants across same test repetitions. 2MWT, Two-Minute Walk Test; DaS, Draw a Shape; DMQ, Daily Mood Questionnaire; IPS, Information Processing Speed; Max., maximum; Min., minimum; SBT, Static Balance Test; UTT, U-Turn Test.

**Supplementary Table S6.** Total number of tests executed, number of participants, tests executed per participant, and range of data generated across active tests and life-space for all evaluable self-declared MS participants.

| **Test** | **Number of tests executed** | **n** | **Number of tests executed per participant** | **Quartiles of number of tests executed** | | | | |
| --- | --- | --- | --- | --- | --- | --- | --- | --- |
|  |  |  |  | **Min.** | **25%** | **50%** | **75%** | **Max.** |
| **DMQ** | 25,718 | 1,291 | 3 | 1 | 3 | 4 | 4 | 5 |
| **IPS Test** | 6,836 | 1,241 | 2 | 0 | 36.5 | 44 | 51 | 75.5 |
| **IPS Digit–Digit Test** | 6,786 | 1,239 | 2 | 0 | 16 | 18 | 20 | 26 |
| **Pinching Test** | 24,930 | 1,227 | 3 | 0 | 21.25 | 30.5 | 41.25 | 69 |
| **DaS Test** | 24,768 | 1,072 | 3 | 0.007 | 0.022 | 0.028 | 0.035 | 0.213 |
| **SBT** | 20,754 | 1,142 | 3 | 0 | 11.08 | 19.79 | 36.745 | 99.25 |
| **UTT** | 18,761 | 901 | 2 | 0.471 | 1.174 | 1.376 | 1.574 | 5.074 |
| **2MWT** | 17,202 | 660 | 2 | 0 | 161 | 206.50 | 231 | 344 |
| **Life-space measurement** | 68,761 | 1,102 | 15 | 0 | 0 | 0 | 4.045 | 165.25 |

Median used per participants across same test repetitions. 2MWT, Two-Minute Walk Test; DaS, Draw a Shape; DMQ, Daily Mood Questionnaire; IPS, Information Processing Speed; Max., maximum; Min., minimum; MS, multiple sclerosis; SBT, Static Balance Test; UTT, U-Turn Test.

**Supplementary Table S7.** Total number of tests executed, number of participants, tests executed per participant, and range of data generated across active tests and life-space for all evaluable self-declared non-MS participants.

| **Test** | **Number of tests executed** | **n** | **Number of tests executed per participant** | **Quartiles of number of tests executed** | | | | |
| --- | --- | --- | --- | --- | --- | --- | --- | --- |
|  |  |  |  | **Min.** | **25%** | **50%** | **75%** | **Max.** |
| **DMQ** | 4,459 | 1,059 | 2 | 1 | 4 | 4 | 4.5 | 5 |
| **IPS Test** | 2,059 | 985 | 1 | 0 | 39 | 47 | 53 | 81 |
| **IPS Digit–Digit Test** | 2,030 | 979 | 1 | 0 | 17 | 20 | 21 | 27 |
| **Pinching Test** | 3,712 | 966 | 2 | 0 | 22.5 | 32.5 | 43.5 | 68 |
| **DaS Test** | 3,542 | 823 | 2 | 0.007 | 0.022 | 0.029 | 0.037 | 0.684 |
| **SBT** | 2,837 | 876 | 1 | 0 | 10.316 | 19.085 | 38.826 | 99.820 |
| **UTT** | 2,371 | 662 | 1 | 0.681 | 1.213 | 1.436 | 1.697 | 7.598 |
| **2MWT** | 1,315 | 337 | 1 | 0 | 98 | 189 | 222 | 382 |
| **Life-space measurement** | 32,428 | 858 | 9 | 0 | 0 | 0 | 1.622 | 82.365 |

Median used per participants across same test repetitions. 2MWT, Two-Minute Walk Test; DaS, Draw a Shape; DMQ, Daily Mood Questionnaire; IPS, Information Processing Speed; Max., maximum; min., minimum; MS, multiple sclerosis; SBT, Static Balance Test; UTT, U-Turn Test.

**Supplementary Table S8.** Differences in persistence in participants using iOS versus Android devices.

| **Persistence definition** | **Self-declared MS participants** | | **Self-declared non-MS participants** | | **All participants** | |
| --- | --- | --- | --- | --- | --- | --- |
|  | Effect size | p-value* | Effect size | p-value* | Effect size | p-value* |
| **At least one full fixed test sequence performed per week** | 0.168 | 0.008 | 0.296 | 0.698 | 0.158 | 0.011 |
| **At least any one test of the fixed sequence performed per week** | 0.108 | 0.625 | 0.627 | 0.024 | 0.127 | 0.299 |
| **At least one 2MWT performed per week** | 0.116 | 0.474 | 0.159 | 0.215 | 0.099 | 0.770 |
| **At least 1 day of life-space measurement data provided per week** | 0.445 | <0.001 | 0.644 | 0.056 | 0.455 | <0.001 |

2MWT, Two-Minute Walk Test; iOS, iPhone Operating System; MS, multiple sclerosis. *Mann–Whitney U test. Green shading to indicate statistical significance.

**Supplementary Table S9.** Differences in the age of participants who persisted for 5 weeks or longer versus participants who persisted for less than 5 weeks.

| **Persistence definition** | **Self-declared MS participants** | | **Self-declared non-MS participants** | | **All participants** | |
| --- | --- | --- | --- | --- | --- | --- |
|  | Effect size | p-value* | Effect size | p-value* | Effect size | p-value* |
| **At least one full fixed test sequence performed per week** | 0.483 | <0.001 | 0.678 | 0.001 | 0.649 | <0.001 |
| **At least any one test of the fixed sequence performed per week** | 0.395 | <0.001 | 0.199 | 0.015 | 0.429 | <0.001 |
| **At least one 2MWT performed per week** | 0.405 | <0.001 | 0.715 | 0.005 | 0.581 | <0.001 |
| **At least 1 day of life-space measurement data provided per week** | 0.327 | <0.001 | 0.203 | <0.001 | 0.302 | <0.001 |

2MWT, Two-Minute Walk Test; MS, multiple sclerosis. *Mann–Whitney U test. Green shading to indicate statistical significance.

**Supplementary Table S10.** Spearman’s rank correlation between maximum break duration and persistence for each of the persistence definitions**.**

| **Persistence definition** | **Self-declared MS participants** | | **Self-declared non-MS participants** | | **All participants** | |
| --- | --- | --- | --- | --- | --- | --- |
|  | Spearman’s rank correlation coefficient | p-value | Spearman’s rank correlation coefficient | p-value | Spearman’s rank correlation coefficient | p-value |
| **At least one full fixed test sequence performed per week** | 0.031 | 0.577 | –0.020 | 0.887 | 0.032 | 0.544 |
| **At least any one test of the fixed sequence performed per week** | 0.262 | <0.001 | 0.670 | <0.001 | 0.318 | <0.001 |
| **At least one 2MWT performed per week** | 0.055 | 0.325 | –0.264 | 0.058 | 0.024 | 0.649 |
| **At least 1 day of life-space measurement data provided per week** | 0.392 | <0.001 | 0.522 | <0.001 | 0.411 | <0.001 |

Spearman’s rank correlation between maximum gap duration and persistence. Green shading to indicate statistical significance. 2MWT, Two-Minute Walk Test; MS, multiple sclerosis.

**Supplementary Table S11.** Association between older age and digital measures (performance on the smartphone-based tests).

| **Test** | **Digital measure** | **Self-declared MS participants** | | **Self-declared non-MS participants** | | **All participants** | |
| --- | --- | --- | --- | --- | --- | --- | --- |
|  |  | RLR model coefficient (95% CI) | p-value | RLR model  coefficient (95% CI) | p-value | RLR model  coefficient (95% CI) | p-value |
|  | **PRO** | | | | | | |
| **DMQ** | Mood score  ([1–5]) | 0.005  (0.001, 0.009) | 0.013 | 0.005  (0.001,  0.009) | 0.012 | 0.005  (0.002,  0.008) | 0.001 |
|  | **Active tests** | | | | | | |
| **IPS Test** | Correct responses | –0.513  (–0.555,  –0.471) | <0.001 | –0.338  (–0.388,  –0.288) | <0.001 | –0.435  (–0.467,  –0.403) | <0.001 |
| **IPS Digit–Digit Test** | Correct responses | –0.157  (–0.170,  –0.144) | <0.001 | –0.116  (–0.129,  –0.102) | <0.001 | –0.138  (–0.148,  –0.129) | <0.001 |
| **Pinching Test** | Successful pinches | –0.343  (–0.404,  –0.282) | <0.001 | –0.172  (–0.243,  –0.101) | <0.001 | –0.267  (–0.314,  –0.221) | <0.001 |
| **DaS Test** | Average best Hausdorff distance across shapes | 0.0002  (<0.001,  <0.001) | <0.001 | <0.001  (<0.001, <0.001) | 0.008 | 0.0001  (<0.001,  <0.001) | <0.001 |
| **SBT** | Sway path, m/s^2^ | –0.093  (–0.330,  –0.145) | 0.444 | –0.307  (–0.555,  –0.059) | 0.015 | –0.190  (–0.362,  –0.017) | 0.031 |
| **UTT** | Average turn speed, rad/s | –0.007  (–0.009,  –0.005) | <0.001 | –0.003  (–0.005,  < –0.001) | 0.031 | –0.005  (–0.007,  –0.004) | <0.001 |
| **2MWT** | Steps | –0.411  (–0.721,  –0.102) | 0.009 | 1.024  (0.573,  1.476) | <0.001 | 0.157  (–0.095,  0.408) | 0.222 |
|  | **Passive monitoring** | | | | | | |
| **Life-space measurement data** | Distance, km | –0.052  (–0.109, 0.004) | 0.070 | –0.010  (–0.108,  0.88) | 0.840 | –0.036  (–0.086,  0.013) | 0.153 |

Green colour indicates statistical significance. A positive robust linear regression coefficient means that older participants had a higher value in the specific assessment feature (e.g. better mood, more IPS correct responses, more pinches, more accurate DaS results, more movement in the SBT, i.e. less balance, higher turning speed in the UTT, more steps in the 2MWT, and larger distance covered in life-space). A negative coefficient means younger participants had a higher value in the specific assessment feature. 2MWT, Two-Minute Walk Test; CI, confidence interval; DaS, Draw a Shape; DMQ, Daily Mood Questionnaire; IPS, Information Processing Speed; MS, multiple sclerosis; PRO, patient-reported outcome; RLR, robust linear regression; s, seconds; SBT, Static Balance Test; UTT, U-Turn Test.

**Supplementary Table S12.** Association between male sex and digital measures (performance on the smartphone-based tests).

| **Test** | **Digital measure** | | **Self-declared MS participants** | | | | **Self-declared non-MS participants** | | | | **All participants** | | |
| --- | --- | --- | --- | --- | --- | --- | --- | --- | --- | --- | --- | --- | --- |
|  |  |  | RLR model coefficient (95% CI) | | p-value | | RLR model coefficient (95% CI) | | p-value | | RLR model coefficient (95% CI) | | p-value |
| **PRO** | | | | | | | | | | | | | |
| **DMQ** | Mood score  ([1–5]) | 0.094  (–0.018, 0.206) | | 0.098 | | 0.055  (–0.046, 0.156) | | 0.286 | | 0.082  (0.008,  0.156) | | 0.030 | |
| **Active tests** | | | | | | | | | | | | | |
| **IPS Test** | Correct responses | –1.229  (–2.357,  –0.100) | | 0.033 | | –0.449  (–1.695, 0.796) | | 0.480 | | –0.785  (–1.621, 0.050) | | 0.065 | |
| **IPS Digit–Digit Test** | Correct responses | –0.318  (–0.671, 0.035) | | 0.077 | | 0.324  (–0.003, 0.650) | | 0.052 | | 0.015  (–0.230, 0.259) | | 0.907 | |
| **Pinching Test** | Successful pinches | 2.632  (0.983, 4.280) | | 0.002 | | 1.572  (–0.211, 3.354) | | 0.084 | | 2.133  (0.918,  3.349) | | 0.001 | |
| **DaS Test** | Average best Hausdorff distance across shapes | 0.002  (<0.001,  <0.001) | | 0.018 | | 0.001  (< –0.001,  0.003) | | 0.141 | | 0.002  (<0.001, 0.003) | | 0.007 | |
| **SBT** | Sway path, m/s^2^ | 6.596  (0.195, 12.997) | | 0.043 | | –0.085  (–6.412, 6.242) | | 0.979 | | 3.289  (–1.249, 7.826) | | 0.155 | |
| **UTT** | Average turn speed, rad/s | –0.009  (–0.061, 0.043) | | 0.735 | | <0.001  (–0.063, 0.063) | | 0.999 | | –0.006  (–0.046, 0.034) | | 0.781 | |
| **2MWT** | Steps | –6.503  (–14.658, 1.652) | | 0.118 | | –18.873  (–33.014,  –4.732) | | 0.009 | | –10.561  (–17.672,  –3.450) | | 0.004 | |
|  | **Passive monitoring** | | | | | | | | | | | | |
| **Life-space measurement data** | Distance, km | –0.545  (–2.008, 0.917) | | 0.465 | | 0.666  (–1.891, 3.224) | | 0.610 | | –0.234  (–1.522, 1.055) | | 0.722 | |

Green colour indicates statistical significance. A positive robust linear regression coefficient means that men had a higher value in the specific assessment feature (e.g. better mood, more IPS correct responses, more pinches, more accurate DaS results, more movement in the SBT, i.e. less balance, higher turning speed in the UTT, more steps in the 2MWT, and larger distance covered in life-space). A negative coefficient means that women had a higher value in the specific assessment feature. 2MWT, Two-Minute Walk Test; CI, confidence interval; DaS, Draw a Shape; DMQ, Daily Mood Questionnaire; IPS, Information Processing Speed; MS, multiple sclerosis; PRO, patient-reported outcome; RLR, robust linear regression; s, seconds; SBT, Static Balance Test; UTT, U-Turn Test.

**Supplementary Table S13.** Demographics of all participants with available quality control data.

| **Variable** | **Participants with available quality control data**  **n=1,917** | **All evaluable participants**  **n=2,483** |
| --- | --- | --- |
| Self-declared MS status, n (%) | 1,082 (56.4) | 1,350 (54.4) |
| Age (years), mean (SD) | 43.0 (12.6) | 42.6 (12.5) |
| Female, n (%) | 1,188 (62.0) | 1,501 (60.1) |
| Height (cm), mean (SD) | 170.1 (16.3) | 170.0 (17.6) |
| Weight (kg), mean (SD) | 78.2 (23.5) | 78.9 (24.3) |
| BMI (kg/m^2^), mean (SD)* | 26.7 (7.8) | 26.8 (8.1) |

BMI, body mass index; MS, multiple sclerosis; SD, standard deviation. *Twenty-three participants (0.93% of all evaluable participants) reporting a height of ≤75 cm who were excluded as entries were deemed to be implausible.

**Supplementary Table S14.**Data collection overview.

| **Data collection** | **Data collected at baseline (first day of use)** | **Data collected during observational period** | **Data collected at study completion/early termination** |
| --- | --- | --- | --- |
| Informed consent | X |  |  |
| Download of Floodlight Open app | X |  |  |
| Self-declared MS status | X |  |  |
| Demographic data (sex, year of birth, country of residence) | X |  |  |
| Weight and height | X |  |  |
| Smartphone-based active tests* | X | X | X |
| Life-space^†^ (passively collected) | X | X | X |

2MWT, Two-Minute Walk Test; DMQ, Daily Mood Questionnaire; GPS, Global Positioning System;
IPS, Information Processing Speed; MS, multiple sclerosis; SBT, Static Balance Test; UTT, U-Turn Test. *Smartphone-based active tests, including DMQ, hand motor function tests (Pinching Test, Draw a Shape Test), gait and postural stability (2MWT, UTT, SBT), and cognition (IPS Test); ^†^Measures mobility levels by recording the distance between the two farthest GPS coordinates detected during the day that are at least 500 metres apart.

**Supplementary Figure S1.** Screenshots of the Floodlight app interface used in this study.


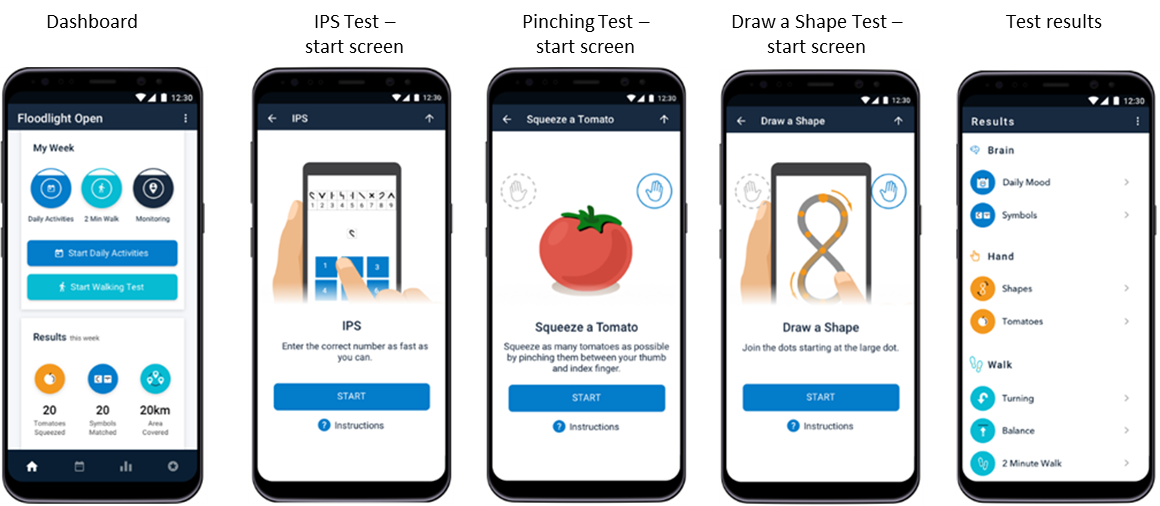


Users could navigate the app via the central dashboard, and easily initiate the different tests and generate results. IPS, Information Processing Speed.
